# Craig Excited-State Aromaticity in Metallabenzenes: How, When, and Why?

**DOI:** 10.1021/jacs.5c18055

**Published:** 2026-02-12

**Authors:** Xuhui Lin, Mingyang Wei, Yirong Mo

**Affiliations:** † Hunan Key Laboratory of Super Microstructure and Ultrafast Process, School of Physics, 12570 Central South University, Changsha, Hunan 410083, China; ‡ Department of Nanoscience, Joint School of Nanoscience and Nanoengineering, 14616 University of North Carolina at Greensboro, Greensboro, North Carolina 27401, United States

## Abstract

Excited-state aromaticity expands the concept of aromaticity to describe additional molecular stability and reactivity upon photoexcitation. While both Hückel and Möbius excited-state aromatic species have been identified, Craig excited-state aromaticity involving [4*n*+2] electrons in planar metallacycles remains unrecognized. Herein, we report that early transition metal (M = Ti, Sc, Y, La, Ac)-based metallabenzenes exhibit Craig 6π aromaticity in their lowest singlet and triplet ππ* excited states, which is supported by a range of aromaticity indices based on electronic, geometric, energetic, and magnetic properties. Notably, *ab initio* valence bond theory reveals that the *d*_*yz*_ orbital dominates the cyclic electron delocalization in the excited-state wave function, resulting in phase inversion between neighboring atomic orbitals of π-symmetry. In contrast, the *d*_*yz*_ orbital is usually doubly occupied in well-identified metallabenzenes with late transition metals which thus display Hückel or Baird (anti)­aromaticity via the *d*_*xz*_ orbital. Our findings provide the first direct evidence and origin of Craig excited-state aromaticity and establish a unified framework for understanding the electronic structure of metallabenzenes, addressing a significant gap in the exploration of excited-state metalla-aromaticity.

## Graphical abstract

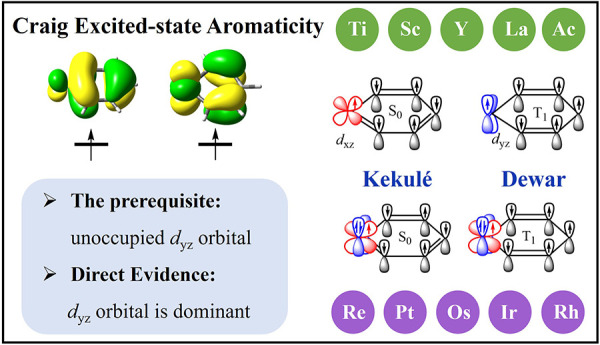

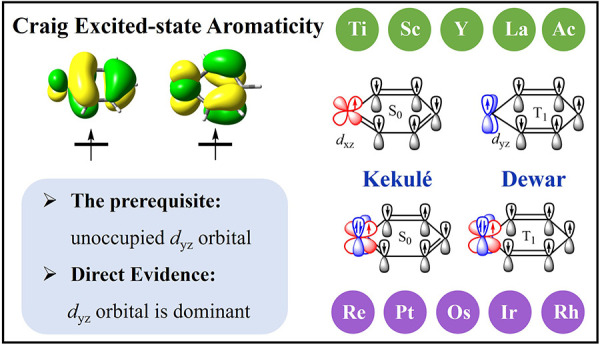

## Introduction

Aromaticity is one of the most essential concepts in chemistry and has been widely recognized as the cornerstone of chemical stability and reactivity.^1–4^ Since Hückel proposed the [4*n*+2] rule for conjugated planar rings in 1931,^5^ various types of aromaticity have been introduced with distinct electron-counting rules.^6^ Remarkably, Craig^7^ and Heilbronner^8^ identified two different types of aromaticity that satisfy the opposite [4*n*] rule in the ground state, both featuring a phase inversion between atomic orbitals of π-symmetry at neighboring atoms. Specifically, Craig-type aromaticity incorporates p_π_–d_π_–p_π_ interactions in planar cyclic molecules, while Heilbronner-type aromaticity arises from twisting the π system into a Möbius strip conformation, namely, Möbius aromaticity. Moreover, the first experimental evidence for Heilbronner-type and Craig-type aromaticity was reported by Herges et al. in 2003 ^9^ and by Xia et al. in 2013,^10^ respectively. Since then, numerous (anti)­aromatic molecules that satisfy these three aromaticity rules in ground states have been reported.^11–14^ Most recently, Pagano et al. and Boronski et al. reported that actinide 2-metallabiphenylenes^15^ and a crystalline trithorium cluster^16^ exhibit Hückel aromaticity, though the simple use of nucleus-independent chemical shift (NICS) to identify aromaticity in heavy-element compounds could be questionable.^17^

While Hückel’s rule governs the singlet ground state of conjugated molecules, Baird extended this framework to excited states, predicting that annulenes with [4*n*]­π electrons exhibit aromaticity in their lowest ππ* triplet states (or triplet ground state).^18–20^ The concept of “excited-state aromaticity”, the evolution of which over time is shown in Figure 1, has since been employed to rationally explain photoinduced structural changes and chemical reactivity in cyclic π-conjugated systems, and it underpins emerging applications in material science.^21–27^ Extensive theoretical and experimental studies on planar and twisted annulenes, transition states, and even all-metal clusters have corroborated Baird’s rule.^28–37^ Additionally, Rzepa and co-workers identified the first stable molecules exhibiting aromaticity in their triplet excited states with [4*n*+2] electrons in a Möbius strip conformation, thus extending Heilbronner-type Möbius aromaticity to the excited states.^38^ Moreover, Karadakov et al. performed a comprehensive computational investigation on the excited-state antiaromaticity for a C_9_H_9_^+^ Möbius ring.^39^

**Figure 1:**
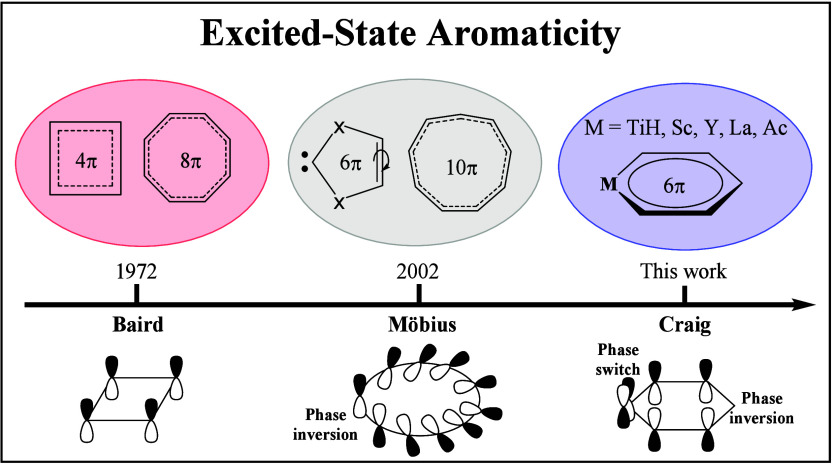
The first discovery of Baird, Möbius, and Craig excited-state aromaticity.

In contrast to the flourishing field of excited-state aromaticity in organic molecules, the identification of excited-state metalla-aromaticity in transition metal compounds is rather limited.^40,41^ The disparity arises primarily from the involvement of *d*-orbitals, which can give rise to either [4*n*+2] Hückel aromaticity or [4*n*] Craig aromaticity in the ground state,^42^ because there are two different topologies of *d*-orbital conjugation within the molecular π-orbitals. As shown in Scheme 1, the combination of the radial *d*_*xz*_ orbital of the metal center with the *p*_*z*_ orbitals of carbon atoms enables traditional Hückel aromaticity. In contrast, the tangential *d*_*yz*_ orbital acts as a “phase switch”, enabling cyclic delocalization to shift to the opposite phase side over the cyclic unit, which leads to Craig aromaticity.^43^ Accordingly, these equivalent *d*-orbitals facilitate the potential for either Baird or Craig (anti)­aromaticity in the excited states. Since both Hückel and Craig molecular orbital (MO) topologies can coexist within the same π-system, it is not always straightforward to determine which topology governs the aromaticity in a given case. Consequently, Szczepanik and Solà suggested that metallacycles exhibit hybrid Hückel–Craig aromaticity.^44^

**Scheme 1:**
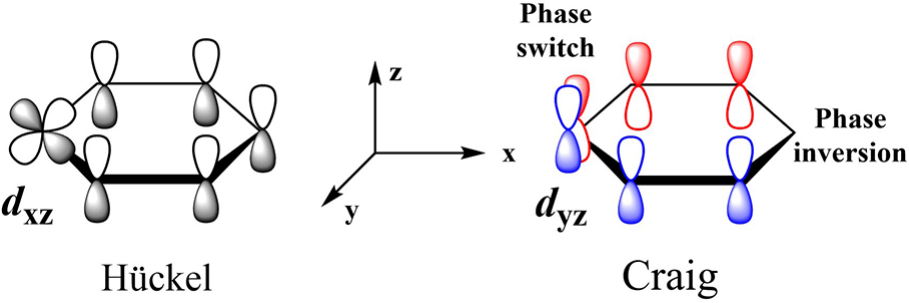
Hückel and Craig Topologies of Molecular Orbitals Involving *d*_*xz*_ and *d*_*yz*_ Orbitals in Metallabenzenes

Given the presence of phase inversion induced by the *d*_*yz*_ orbital, Craig aromaticity is usually considered a planar type of Möbius aromaticity.^43–45^ Alternatively, it can also be regarded as a variant of Hückel or Baird aromaticity in which one or more *p* orbitals are replaced by *d*_*yz*_ orbitals. Because a transition metal typically contributes two *d* orbitals to the π-conjugation, Craig aromaticity extends beyond the standard Hückel molecular orbital (HMO) theory,^5^ mainly due to the multiple *d*–*p* bonding interactions, while both Hückel and Heilbronner–Möbius aromaticity can be well-defined within the conventional HMO framework. Despite considerable theoretical and experimental progress in identifying Craig (anti)­aromatic systems,^46–48^ a comprehensive and unified theoretical foundation for it remains elusive. As a result, there is no consensus whether Craig aromaticity is a direct extension of Hückel aromaticity or a planar type of Möbius aromaticity.^49^ Nonetheless, the essential role of *d* orbitals (usually the *d*_*yz*_ orbital) fundamentally distinguishes Craig aromaticity from that of both classical frameworks. Therefore, the term “Craig aromaticity” is more appropriate to describe aromaticity dominated by the *d*_*yz*_ orbital, particularly for systems following an electron-counting rule opposite that of Hückel or Baird aromaticity. In this context, “Möbius” should be simply used to highlight the phase inversion induced by the *d*_*yz*_ orbital rather than to define the aromaticity type.

Although Craig pointed out that *d*-orbital-involved planar systems exhibit aromatic character without a preference for [4*n*+2] or [4*n*] electrons,^7^ most of the reported aromatic metallacycles follow Hückel’s rule. Moreover, to the best of our knowledge, all reported excited-state metalla-aromatics so far follow Baird’s rule. That is to say, “*there are still no examples of excited-state aromatic Craig-type Möbius-aromatic compounds in the literature*.” ^50^ Recently, we reported that planar four-membered actinides with 4π and 4σ electrons can exhibit double Craig aromaticity in the ground state, which is also observed in closed-shell and open-shell transition metal counterparts.^51–54^ This suggests that six-membered metallacycles containing 6π electrons could be promising candidates for Craig excited-state aromaticity. In this regard, metalla­benzenes are exemplary six-membered metallacycles formed by substituting one CH group of benzene with a transition metal fragment.^55–57^ Although well-identified metallabenzenes are generally Hückel aromatic in the ground states,^58–60^ their electronic structures offer a unique opportunity to explore the possibility of Craig excited-state aromaticity.

In this work, we present a comprehensive theoretical investigation of the (anti)­aromatic nature of metallabenzenes incorporating both early and late transition metals in their lowest ππ* excited states. Surprisingly, early transition metal (ETM)-based metallabenzenes with explicit 6π electrons exhibit Craig excited-state aromaticity, whereas their late transition metal (LTM) counterparts typically display Baird (anti)­aromaticity. Furthermore, *ab initio* valence bond (VB) theory provides direct evidence and mechanistic insight into the Craig excited-state aromaticity of metallabenzenes.

## Computational Details

All spin-unrestricted DFT (UDFT) and time-dependent DFT (TDDFT) calculations with the PBE0 functional^61^ augmented with Grimme’s D3­(BJ) dispersion correction^62^ were performed with Gaussian 16,^63^ while complete active space self-consistent field (CASSCF) and the subsequent second-order perturbation theory (CASPT2) computations were conducted with the OpenMolcas software.^64^ The NICS values at the CASSCF level were computed with Dalton software,^65^ where the MOKIT program was used to prepare the initial guesses from the Gaussian- and OpenMolcas-generated MOs.^66^ Basis sets with the Stuttgart effective-core potentials were employed for transition metals,^67–69^ while main group elements adopted the def2-TZVPP basis set.^70^ For actinides, the small-core fully relativistic effective core potential and associated segmented valence basis sets (ECP60MDF)^71^ were adopted. All VB calculations (see details in the Supporting Information), including VBSCF,^72^ BLW, and SCGVB, were performed with XMVB software.^73,74^ Additionally, we also performed computations for the TiHC_5_H_5_ with the def2-QZVP basis set, yielding results that were nearly identical to those obtained with effective-core potentials.

## Results and Discussion

### I The Presence of Craig Excited-State Aromaticity in ETM-Based Metallabenzenes: How and When?

#### MO Perspective on the Craig Excited-State Aromaticity

We first study the ETM-based metallabenzenes MC_5_H_5_ (M = TiH, Sc, Y, La, and Ac) with six delocalized π-electrons, because the transition metal fragments share a common valence electronic configuration (*d* ^1^ *s* ^2^). To unravel their electronic structures, we perform CASSCF/PT2­(6e,7o) calculations, where the seven active MOs arise from the linear combinations of two *d* orbitals (*d*_*xz*_ and *d*_*yz*_) from the metal center and five carbon *p*_*z*_ orbitals from the C_5_H_5_ moiety (see Figure S1). As shown in Figure 2a, the multireference computations show that the ground state (S_0_) of each planar MC_5_H_5_ with *C*_2*v*_ symmetry is a singlet ^1^ *A*_1_, while the lowest singlet (S_1_) and triplet (T_1_) ππ* excited states correspond to ^1^ *B*_2_ and ^3^ *B*_2_, respectively.

**Figure 2:**
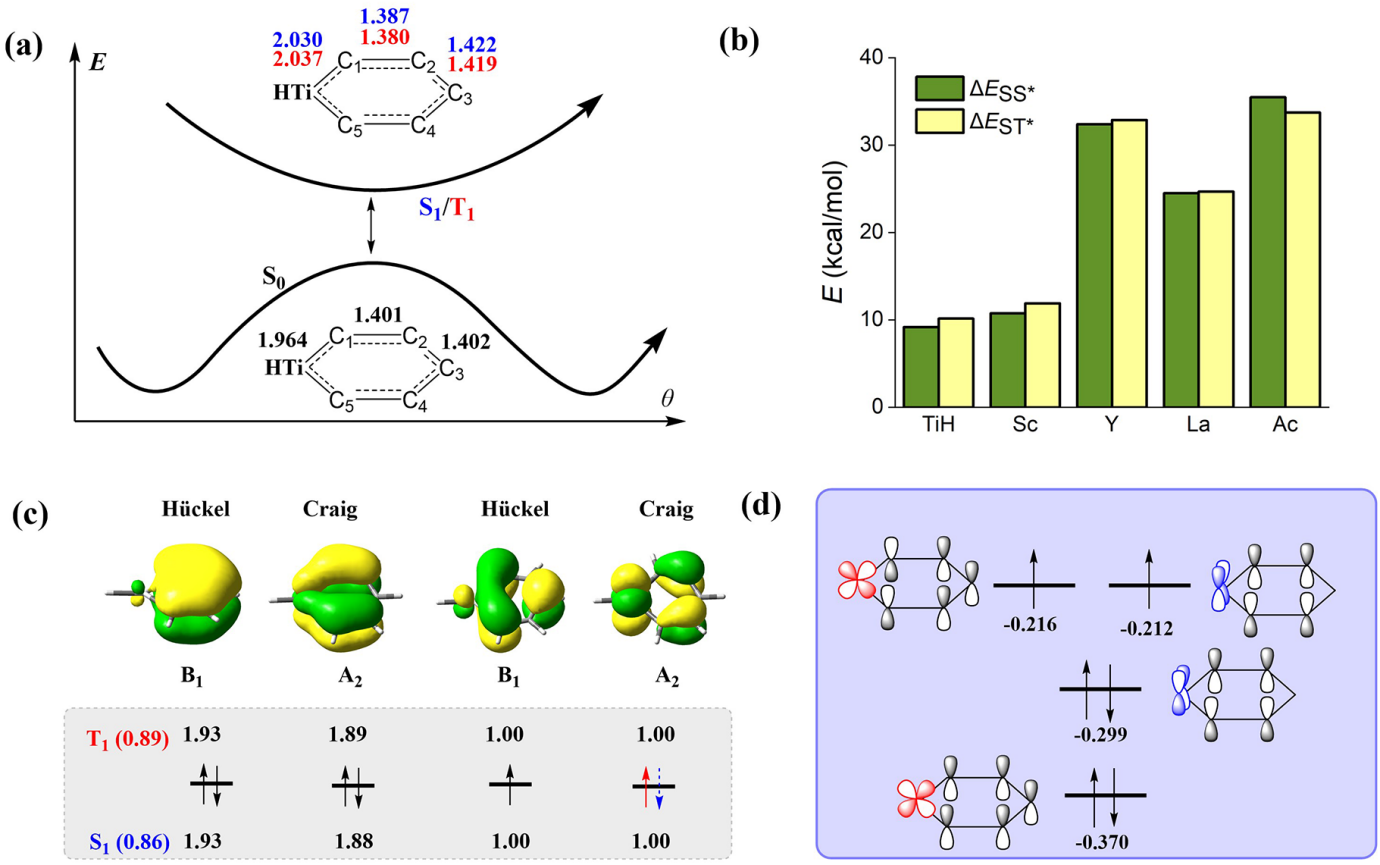
(a) Qualitative illustration of the potential energy curves as a function of the dihedral angle θ_C2‑C1‑M‑C5_, where the optimal bond distances (in Å) refer to the lowest singlet (S_1_, blue data) and triplet (T_1_, red data) ππ* excited states and singlet ground state (S_0_) of TiHC_5_H_5_ by the CASPT2 method. (b) Adiabatic excitation energies for the S_1_ (Δ*E*_SS*_) and T_1_ (Δ*E*_ST*_) states at the CASPT2 level, for which the data by DFT and VBSCF can be found in Table S2. (c) The four main active molecular orbitals (isovalue = 0.03) and their occupation numbers, along with the weights for the most important configurational state function (CSF) for the T_1_ and S_1_ states of TiHC_5_H_5_ by the CASSCF­(6e,7o) method. (d) MO (in a.u.) diagram for the T_1_ state of TiHC_5_H_5_ at the UDFT level.

Interestingly, both CASSCF and DFT methods reveal that the planar ^1^ *A*_1_ state is unstable and appears to be a transition state, which would transition to a twisted geometry featuring a Dewar-like structure, consistent with the literature.^75,76^ Moreover, the most stable isomer of TiHC_5_H_5_ is found to be an open-sandwich hydride structure. Accordingly, TiHC_5_H_5_ is predicted to be antiaromatic, although the planar structure exhibits identical C–C bond distances. While aromaticity usually provides extra thermostability to conjugated systems due to cyclic electron delocalization, it is also associated with less stable or even unstable structures, such as transition-state aromaticity.^77^

In sharp contrast, the lowest ππ* triplet (T_1_) and singlet (S_1_) excited states can remain stable with a planar conformation, suggesting that the S_1_ and T_1_ states may exhibit aromaticity due to extra thermostability. Notably, the standard UDFT will not always generate ππ* triplets directly, except for TiHC_5_H_5_, because the lowest triplet states may correspond to πσ* states, for which the ΔSCF procedures should be employed. It can be seen from Figure 2b that the singlet–triplet energy gaps are approximately 10 kcal/mol for light metallabenzenes and increase to around 30 kcal/mol for heavy metallabenzenes. Interestingly, the CASSCF wave function indicates that both S_1_ and T_1_ states share similar electronic configurations, with the primary difference being the spin directions of the unpaired electrons. Therefore, the optimal geometries for S_1_ and T_1_ states of all metallabenzenes should be nearly identical, as evidenced by the CASPT2 results (see Figure 1a).

Moreover, the most important configurational state function (CSF) consists of four main active orbitals, including two doubly occupied MOs and two singly occupied MOs (SOMOs), as shown in Figure 2c, consistent with Kohn–Sham canonical MOs (CMOs, in Figure 2d). Among them, the A_2_ SOMO is characterized with bonding in C_1_–C_2_ and C_4_–C_5_, resulting in minor C–C bond length alteration (BLA) for the equilibrium geometries, suggesting that the excited states of metallabenzenes seem to be dominated by the Dewar resonance structure. It should be noted that the shapes of the Kohn–Sham MOs do not guarantee cyclic delocalization because the validity of Koopman’s theorem is limited to the HOMO at the DFT level.^78^ Therefore, we obtained localized CASSCF active orbitals through the Pipek–Mezey localization^79^ (see Figure S2), which confirm the cyclic delocalization of the π-orbitals.

Because the weights of the most important CSF for the S_1_ and T_1_ states are close to 0.9 (e.g., 0.86 and 0.89 for TiHC_5_H_5_), the DFT methods can provide reliable optimal geometries comparable to those obtained with CASSCF and CASPT2 methods. In this regard, TDDFT and UDFT computations with the PBE0 functional were further conducted to optimize the S_1_ and T_1_ geometries, respectively, and the major optimal bond distances are listed in Table 1. For comparison, we also performed TDDFT computations to obtain the optimal geometries for T_1_ states (see Table S1). As expected, the optimal geometries are nearly identical for S_1_ and T_1_ states with both multireference and DFT methods. In the following, we adopted the DFT geometries and results (TDDFT for S_1_ and UDFT for T_1_) for discussion, unless mentioned otherwise. Since there are six electrons but seven π-MOs, it is obvious that the two unpaired π-electrons in the T_1_ state would occupy two near-degenerate π-MOs, and this electron configuration would exhibit 6π aromaticity. This is consistent with the MO diagram at the UDFT level for the T_1_ state of TiHC_5_H_5_ (Figure 2d), where the two SOMOs are quasi-degenerate. However, the two SOMOs are not always near-degenerate for the remaining metallabenzenes.

**Table 1:** Optimal Bond Distances (in Å), NICS(1)_*zz*_ Values (in ppm), and Induced Ring Current Density (*J* ^ind^, in nA/T) for the Lowest Singlet (S_1_, TDDFT) and Triplet (T_1_, UDFT) ππ* States of Metallabenzenes with the PBE0 Functional and Their Resonance Energy (RE, in kcal/mol) with the VBSCF Method

Molecule	State	M–C_1/5_	C_1_–C_2_	C_3_–C_4_	NICS(1)_*zz*_	NICS(1)_*zz*_ ^a^	*J*_ind_	MCI	RE
TiHC_5_H_5_	T_1_	2.049	1.373	1.415	–26.2	–51.5	9.2	0.504	26.5
	S_1_	2.015	1.384	1.410	/	–49.2	/	0.393	26.1
ScC_5_H_5_	T_1_	2.136	1.378	1.416	–33.3	–28.3	11.6	0.454	24.2
	S_1_	2.109	1.384	1.413	/	–31.4	/	0.375	28.0
YC_5_H_5_	T_1_	2.256	1.384	1.415	–28.3	–15.3	12.0	0.451	25.0
	S_1_	2.253	1.385	1.413	/	–22.4	/	0.402	30.1
LaC_5_H_5_	T_1_	2.369	1.380	1.414	–22.3	–28.5	11.1	0.461	26.2
	S_1_	2.364	1.381	1.412	/	–45.7	/	0.370	32.2
AcC_5_H_5_	T_1_	2.447	1.381	1.415	–24.2	–41.6	11.3	0.412	25.2
	S_1_	2.445	1.382	1.414	/	–39.9	/	0.395	31.6
C_6_H_6_	S_0_	/	1.387	1.387	–30.9	–27.2	12.0	0.666	24.5

a NICS­(1)_*zz*_ values obtained with the CASSCF method.

NICS is one of the most widely accepted aromaticity criteria for planar rings,^80^ and its negative or positive values indicate aromaticity or antiaromaticity, respectively. In particular, NICS(1)_*zz*_ is the most effective NICS index that can be easily computed for both S_0_ and T_1_ states.^81,82^ Currently, the NICS value for the singlet excited state cannot be directly evaluated with the DFT method. Instead, Wu and Ottosson proposed an approximate approach to estimate the NICS(1)_*zz*_ values for S_1_ states based on NICS calculations of open-shell triplet states using the optimal geometries of S_1_ states.^25^ But this approach seems oversimplified, and its applicability is likely limited to molecules with low symmetry where the singlet excited state shares the same state symmetry as the corresponding triplet excited state. Alternatively, the NICS values for excited states can be effectively computed by the CASSCF method^83,84^ with Dalton software.^65^ In this respect, Karadakov et al. presented many helpful and comprehensive studies on the excited-state aromaticity in hydrocarbon systems.^39,85,86^ As shown in Table 1, the NICS(1)_*zz*_ values are all negative in both the S_1_ and T_1_ states, supporting the presence of 6π excited-state aromaticity in ETM-based metallabenzenes. It is worth noting that the NICS values using the CASSCF method are very sensitive to the wave function and active space in transition metal compounds.

We continued to apply the gauge including magnetically induced current (GIMIC)^87^ method to derive the induced current density (*J* ^ind^) for T_1_ states at the UDFT level, which was integrated over a square plane 8 Å away from the center of a ring along the axis of the M–C bond, spanning 5 Å both above and below the plane of the ring. The values of *J* ^ind^ for T_1_ states range from 9 to 12 nA/T and are comparable to the value for benzene in the ground state (11.0 nA/T). Furthermore, the computed multicenter bond indices (MCIs) using Multiwfn^88^ for S_1_ (UDFT) and T_1_ (TDDFT) states of ETM-metallabenzenes are also comparable to that of the ground state of benzene. Thus, all of the NICS values, *J* ^ind^, and MCI support the presence of 6π excited-state aromaticity in ETM metallabenzenes.

Given that the ETM-based metallabenzenes explicitly contain six delocalized π electrons, the excited-state aromaticity should be classified as Craig-type rather than Baird-type aromaticity. Notably, it has been shown that the numbers of π-CMOs with Hückel topology (*d*_*xz*_ orbital) and Craig–Möbius topology (*d*_*yz*_ orbital) are usually the same in Craig aromatic systems, such as osmapentalynes,^10^ ReB_4_^–^,^89^ and Pa_2_B_2_.^51^ In ETM-based metallabenzenes, there are indeed one with Hückel topology and the other with Craig–Möbius topology in both doubly occupied and singly occupied π-CMOs, suggesting that they may exhibit Craig excited-state aromaticity. However, there is no clear correlation between the number of Hückel-type or Craig-type MOs and the aromatic nature, though Mauksch and Tsogoeva presented a rule to classify metallacycles as either Hückel-type or Craig-type aromaticity based on the number of total π-MOs versus the number of Craig-type MOs.^45^ While both Hückel-type and Craig-type MOs can coexist within the Craig aromatics, only Heilbronner–Möbius MOs are present in twisted Möbius aromatics. This is also consistent with the Heilbronner–Möbius excited-state aromaticity, in which the MOs of the T_1_ state for twisted C_4_OF_4_ are exclusively of Heilbronner–Möbius topology (see Figure S4).

Furthermore, the delocalized spin density for T_1_ states of ETM-based metallabenzenes (see Figure S3) mainly arises from the *d*_*yz*_ orbital, further suggesting the presence of Craig excited-state aromaticity. However, it is difficult to state with certainty whether the metallabenzenes exhibit Craig or Baird excited-state aromaticity based solely on MOs, because both *d*_*xz*_ and *d*_*yz*_ orbitals contribute to electron delocalization. It is also worth noting that the T_1_ states of ClTiC_5_H_5_ and CH_3_TiC_5_H_5_, along with MHC_5_H_5_ (M = Zr, Hf, and Th), share similar geometric, electronic, and aromatic characters with TiHC_5_H_5_ (see details in Table S3), except that MHC_5_H_5_ (M = Zr, Hf, and Th) are transition states. The intrinsic reaction coordinate (IRC) calculations at the UDFT level further reveal that the planar MHC_5_H_5_ would transition to the minima with the hydrogen atom bonded to the metal center deviating from the M–C_5_H_5_ plane.

#### VB Perspective on the Craig Excited-State Aromaticity

To seek an improved understanding of the electron delocalization and related aromaticity in ETM-based metallabenzenes, we resorted to *ab initio* valence bond (VB) theory, where the many-electron wave function is constructed by a series of localized VB (or resonance) structures.^90–93^ Here, the VBSCF­(6e,7o) calculations were adopted, with seven active atomic orbitals referring to five carbon *p*_*z*_ orbitals and two metal *d* orbitals (*d*_*xz*_ and *d*_*yz*_) (see Figure S5). The VBSCF calculations using both full and covalent structure sets show that S_0_, S_1_, and T_1_ state wave functions are predominantly composed of three major VB structures, including one Dewar structure and two Kekulé structures, as illustrated in Figure 3a. The weights of VB structures for different VB sets are given in Table S4. In the following, we adopted the VBSCF results using the covalent structure set, unless otherwise noted.

**Figure 3:**
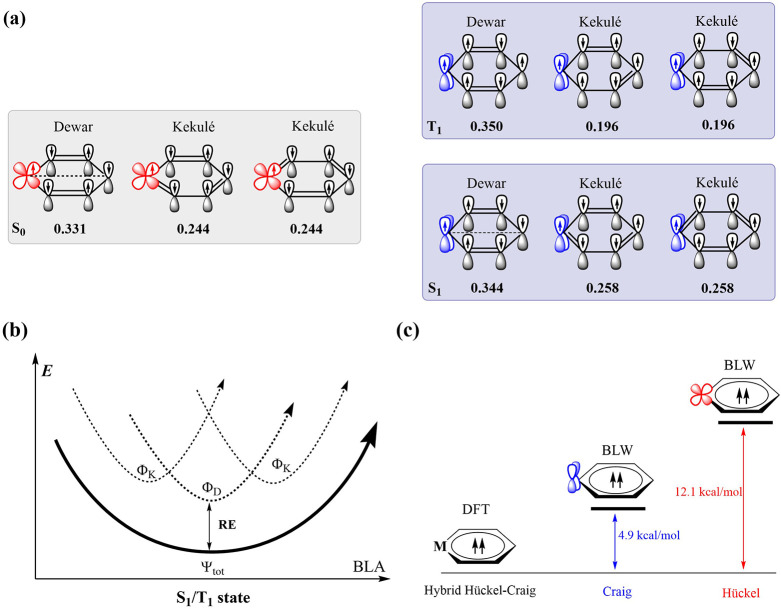
(a) The three major VB structures and their structural weights of S_0_, S_1_, and T_1_ states for TiHC_5_H_5_. (b) Schematic potential energy curves as a function of the bond distance alteration (BLA) for the S_1_ or T_1_ state (Ψ_tot_) in the planar conformation and the corresponding Dewar (Φ_D_) and Kekulé (Φ_K_) resonance structures. (c) The T_1_ state of TiHC_5_H_5_ with conventional DFT and BLW methods, where the mixed *d*_*xz*_–*d*_*yz*_ and only *d*_*xz*_ or *d*_*yz*_ orbitals are considered in cyclic electron delocalization.

In contrast to benzene,^94^ the Dewar structure significantly contributes to the S_0_ state of metallabenzenes with a structural weight around 0.331, and the weight of the Dewar structure is larger than that of a single Kekulé structure, typically in light metallabenzenes. The preference of the Dewar structure in the S_0_ state is consistent with the previous finding that the ground state is a transition state and would transit to a nonplanar geometry featuring a Dewar-like structure.^75,76^ Moreover, we also performed spin-coupled generalized valence bond (SCGVB)^95–98^ calculations for TiHC_5_H_5_ using overlap enhanced orbitals, which have been applied to highlight the strong dominance of Kekulé structures in heteroaromatic molecules.^99^ The SCGVB results show that the weight for a single Kekulé structure reaches 0.276, while the weight for a single Dewar structure is 0.222.

As for T_1_ and S_1_ excited states, it is the Dewar structure rather than any Kekulé structure that determines the excited-state wave function, which is consistent with the minor bond length alternation (BLA) observed in the equilibrium geometries by DFT and CASPT2 methods. Thus, three VB structures are required to accurately describe the excited-state wave functions, as depicted in the schematic potential energy surface (PES) in Figure 3b. Furthermore, we evaluated the resonance energy (RE) of excited states by extracting the energy of a specific resonance structure (the Dewar structure for metallabenzenes) from the total energy of the system using the VBSCF­(6e,7o) method. As shown in Table 1, the computed RE for both S_1_ and T_1_ state metallabenzenes is approximately 25–30 kcal/mol, comparable to the resonance energy of benzene (24.5 kcal/mol) in the ground state. This highlights significant cyclic electron delocalization in the excited states of ETM-based metallabenzenes. However, it is crucial to note that cyclic electron delocalization is not directly associated with aromaticity, as it occurs in both aromatic and antiaromatic systems. Moreover, even the presence of large resonance energy does not necessarily result in aromaticity.^100^ The key difference is that cyclic electron delocalization provides extra stability to aromatic systems but induces instability in antiaromatic systems when compared to their acyclic references. In this context, the extra cyclic resonance energy (ECRE), defined as the resonance energy difference between a cyclic compound and its appropriate acyclic reference, serves as a straightforward index for (anti)­aromaticity,^101–103^ which is discussed in the following section.

More importantly, the VBSCF computations indicate that the *d*_*yz*_ orbital dominates the S_1_ and T_1_ excited-state wave functions, whereas the *d*_*xz*_ orbital is responsible for the ground state. Notably, the VB structures with *d*_*xz*_ or *d*_*yz*_ orbitals also contribute to the excited- or ground-state wave function, though their weights are insignificant. This suggests that the excitation process can be interpreted as an electron being excited from *d*_*xz*_ to *d*_*yz*_. The preference for the *d*_*y*__*z*_ orbital in the excited-state wave function, which results in the phase inversion of the overlapping π_*d‑p*_ electron delocalization, provides direct evidence for Craig 6π excited-state aromaticity. In this regard, the block-localized wave function (BLW) method,^104–107^ which is the simplest variant of *ab initio* VB theory that incorporates MO or DFT computational efficiency, presents a promising approach for identifying Hückel or Craig aromaticity in transition metal compounds. This is because the BLW method can quantitatively derive the wave function for a diabatic state, where only one *d* orbital is allowed to participate in electron delocalization at the DFT level. As shown in Figure 3c, the Craig state with the *d*_*yz*_ orbital is approximately 7 kcal/mol more stable than the Hückel state with the *d*_*xz*_ orbital, confirming the preference for the *d*_*yz*_ orbital in excited-state aromaticity. Moreover, the BLW method can provide geometric and orbital information for isolated Craig and Hückel structures, which is currently under development.

Our VB results suggested that the excited-state aromaticity in metallacycles has a hybrid Hückel–Craig nature, similar to the ground-state metalla-aromaticity.^44^ In particular, the excited-state aromaticity in ETM-based metallabenzenes can be exclusively classified as Craig aromaticity since the Craig contribution significantly outweighs the Hückel one.

#### Magnetic and Energetic Criteria for Craig Excited-State Aromaticity

Most recently, Orozco-Ic et al. proposed that the core electrons may have a significant impact on the magnetic properties, such as NICS and *J* ^ind^, particularly for heavy-element clusters.^108^ To exclude the role of the core electrons of the metal center on the NICS value, we compared the NICS(1)_*zz*_ values (see Table S5) for all MHC_5_H_5_ (M = Ti, Zr, Hf, and Th) and MC_5_H_5_ (M = Sc, Y, La, and Ac) with their saturated analogues H_2_MC_5_H_10_ and HMC_5_H_10_. All of the NICS values for the saturated analogues are around zero, indicating that they are nonaromatic and NICS is at least a reliable probe for aromaticity in this study. Moreover, the plotted NICS(1)_*zz*_ grids in the molecular plane for the T_1_ state of TiHC_5_H_5_ (see Figure 4a) clearly show that the NICS(1)_*zz*_ values are consistently negative throughout the entire ring but become much more positive around the metal center. Accordingly, the core electrons of the metal center have a relatively insignificant effect on the magnetic response in the studied metallabenzenes.

**Figure 4:**
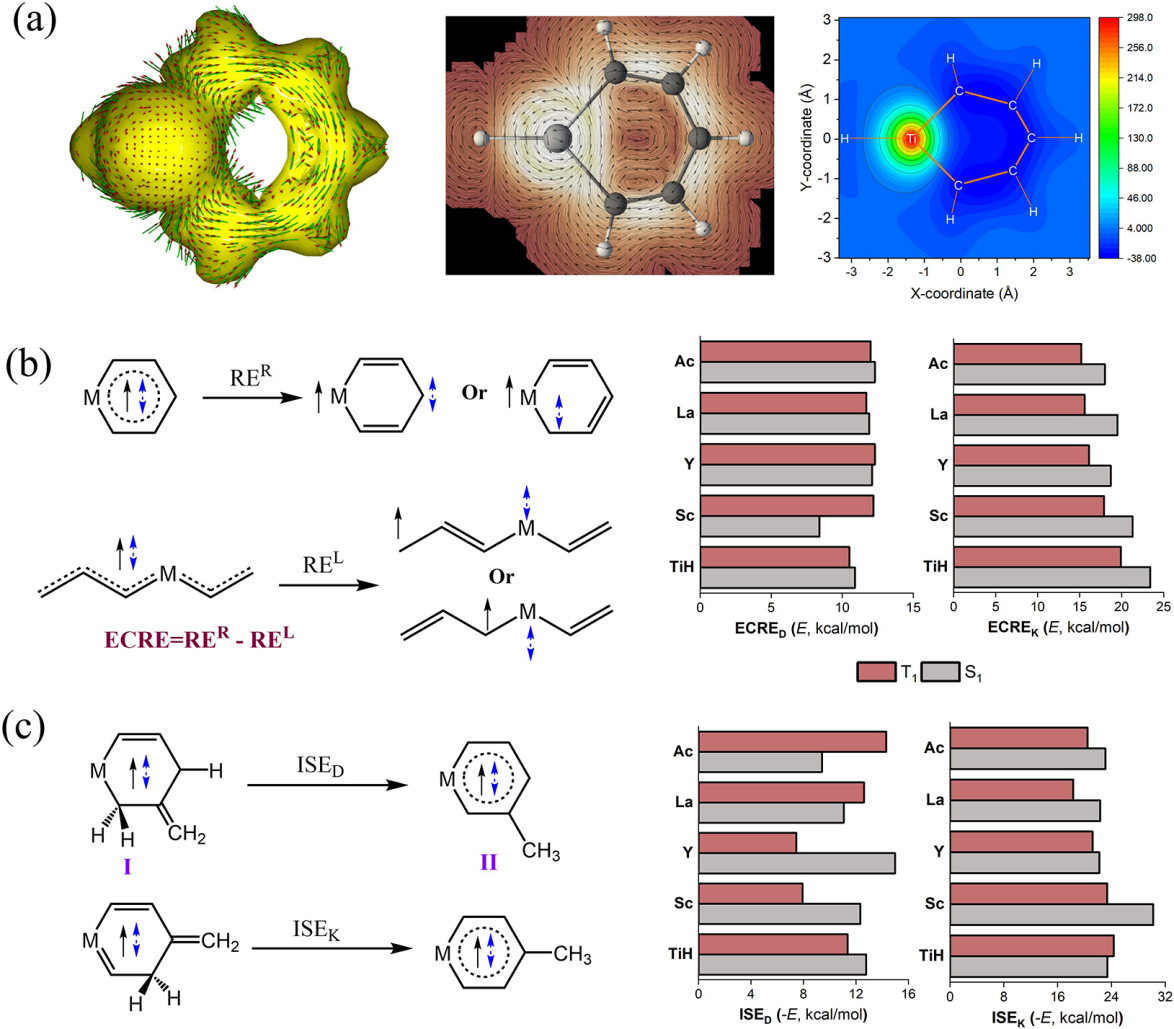
(a) ACID plots, induced current at 1.5 Bohr above the molecular plane by the GIMIC program, and NICS(1)_*zz*_ scan for the T_1_ state of TiHC_5_H_5_. Evaluation of (b) extra cyclic resonance energy (ECRE) and (c) isomerization stabilization energy (ISE) for the S_1_ and T_1_ states of metallabenzenes at the VBSCF and DFT (UDFT and TDDFT for the T_1_ and S_1_ states, respectively) levels.

Furthermore, we plotted the isosurfaces of anisotropy of the induced current density (ACID)^109^ and the line integral convolution visualization of the induced current by GIMIC method. As shown in Figure 4a, both ACID and GIMIC plots clearly depict diatropic ring currents around the ring and paratropic local currents around the metal centers, further supporting the 6π excited-state aromaticity. The ACID plots for the remaining metallabenzenes can be found in Figure S6. Notably, Sundholm and co-workers found that the ring current does not pass from one side of the molecular plane to the other in osmapentalenes and osmapentalynes, which is expected in the Craig (anti)­aromatics.^49^ Moreover, the ring current even avoids passing through the metal center. This phenomenon is also observed in our cases, where the ring currents flow through the inner region of the ring. There are two main reasons why the ring current avoids the metal center. First, the metal center exhibits strong local circulation with tens of core electrons, whereas the ring current arises from six delocalized valence electrons. Consequently, when the ring current attempts to pass through the metal center, the strong local circulation may push it toward the inner region of the ring. Second, the metal center typically exhibits strong spin–orbit coupling, which can influence the ring current around the metal center.

While magnetic indices may spark debates in heavy-element systems due to the complex behavior of core electrons,^17,110,111^ electronic and energetic indices may serve as more reliable indices for probing aromaticity, because they focus on the delocalized valence electrons and thus inherently exclude the contributions from core electrons, as suggested by Orozco-Ic et al.^108^ Nevertheless, for those aiming to explicitly study the role of core electrons in magnetic response and aromaticity, the method proposed by Orozco-Ic et al.^108^ offers a useful and well-established approach.

In particular, ECRE, as mentioned above and originally proposed by Dewar,^112^ directly measures the thermostability induced by cyclic electron delocalization, serving as a convincing indicator of (anti)­aromaticity. Specifically, a positive ECRE quantifies aromaticity, while a negative ECRE corresponds to an antiaromatic system. The ECRE for a nonaromatic system is expected to be close to zero. Here, we adopted linear M­(CH_2_)_2_(CH)_3_ (see Figure 4b) as acyclic references, which similarly involve six active electrons and seven active orbitals. VBSCF­(6e,7o) calculations for linear systems revealed that two major VB structures are dominant in the excited-state wave functions, one referring to a variant of Dewar structure and the other to a Kekulé variant. The structural weights of these two VB structures are 0.47 and 0.53 for light metallabenzenes TiHC_5_H_5_ and ScC_5_H_5_, but they shift to 0.54 and 0.46 for the remaining heavy metallabenzenes. Therefore, we derived both ECRE_D_ and ECRE_K_ by comparing the resonance energy difference between cyclic metallabenzenes (RE^R^) and their linear references (RE^L^) with respect to the Dewar and Kekulé structures, respectively. It was found that ECRE_D_ and ECRE_K_ are positive for both T_1_ and S_1_ states of all ETM-based metallabenzenes, confirming the Craig 6π excited-state aromaticity.

In addition, we also employed the popular isomerization method^113,114^ to assess the excited-state aromaticity by comparing the energy difference between a localized isomer I and a delocalized isomer II at the DFT level, both of which exist in planar conformations under *C*_*s*_ symmetry (Figure 4b). Notably, the similar spin density plots for delocalized isomer II with unsubstituted metallabenzenes (Figure S3) confirm that the spin density is delocalized across the ring. We adopted two types of strategies to evaluate the isomerization stabilization energy (ISE) based on Dewar and Kekulé resonance structures, respectively. As expected, the negative values of ISE_D_ and ISE_K_ for both the T_1_ and S_1_ states support the 6π excited-state aromaticity in ETM-based metallabenzenes.

Given that VBSCF demonstrates that the *d*_*xz*_ orbital dominates the electron delocalization in the ground state of ETM-based metallabenzenes, they should be 6π aromatic, satisfying Hückel’s rule. The ground-state aromaticity is also supported by geometric and energetic aromaticity indices, including equal C–C bond distances (see Figure 2a), ISE (−11.0 kcal/mol for TiHC_5_H_5_), and ECRE (12.9 kcal/mol), despite the NICS(1)_*zz*_ values being positive (92.4). This suggests again that magnetic indices may lead to the misinterpretation of aromaticity.

### II The Absence of Craig Excited-State Aromaticity in LTM-Based Metallabenzenes: Why?

From the above context, it is evident that ETM-based metallabenzenes can exhibit Craig 6π excited-state aromaticity. However, most well-identified metallabenzenes typically involve late transition metals (LTM), for which the exact number of π electrons responsible for aromaticity remains uncertain.^115^ Thorn and Hoffmann originally proposed that metallabenzenes are 6π-aromatic,^55^ while Schleyer and Wang argued that eight delocalized π electrons contribute to aromaticity.^42^ Moreover, Fernández and Frenking suggested that metallabenzenes could even be 10π-electron aromatic systems, based on a derivative of rhodabenzene.^59^ Despite these varying interpretations, LTM-based metallabenzenes are generally believed to exhibit Hückel and Baird (anti)­aromaticity in their ground and excited states, respectively. Therefore, it is essential to understand why Craig excited-state aromaticity emerges in ETM-based metallabenzenes but not in LTM-based ones.

We first examined rhenabenzene Re­[C_5_H_5_]­(CO)_4_,^116^ which is known to be aromatic in the ground state with an ISE of 21.5 kcal/mol.^60^ DFT calculations reveal that there are eight π electrons forming four CMOs (Figure 5a), suggesting Craig-type rather than Hückel-type aromaticity. It should be noted that the HOMO of (CO)_4_ReC_5_H_5_ refers to the lone-pair electrons mainly localized on the *d*_*x* ^2^‑*y* ^2^_ orbital of the metal center. Interestingly, the HOMO–1 shows antibonding character between the metal *d*_*yz*_ orbital and the C_5_H_5_ π-system, while the remaining three low-lying π-CMOs resemble those found in TiHC_5_H_5_. A possible π-orbital interaction scheme is shown in Figure 5b, where the *d*_*yz*_ orbital from the transition metal fragment (CO)_4_Re^+^ is doubly occupied. As a result, HOMO–1 and HOMO–4 can be interpreted either as bonding/antibonding combinations between *d*_*yz*_ and the C_5_H_5_ moiety or alternatively as an occupied *d*_*yz*_ orbital and an occupied π orbital primarily localized on C_5_H_5_. In other words, the lone pair on *d*_*yz*_ can be understood as a bystander, playing a destabilizing role in the π conjugation due to Pauli repulsion. In the meantime, *d*_*xz*_ participates in the ring conjugation. Ultimately, rhenabenzene should exhibit 6π Hückel aromaticity, despite having four π-CMOs.

**Figure 5:**
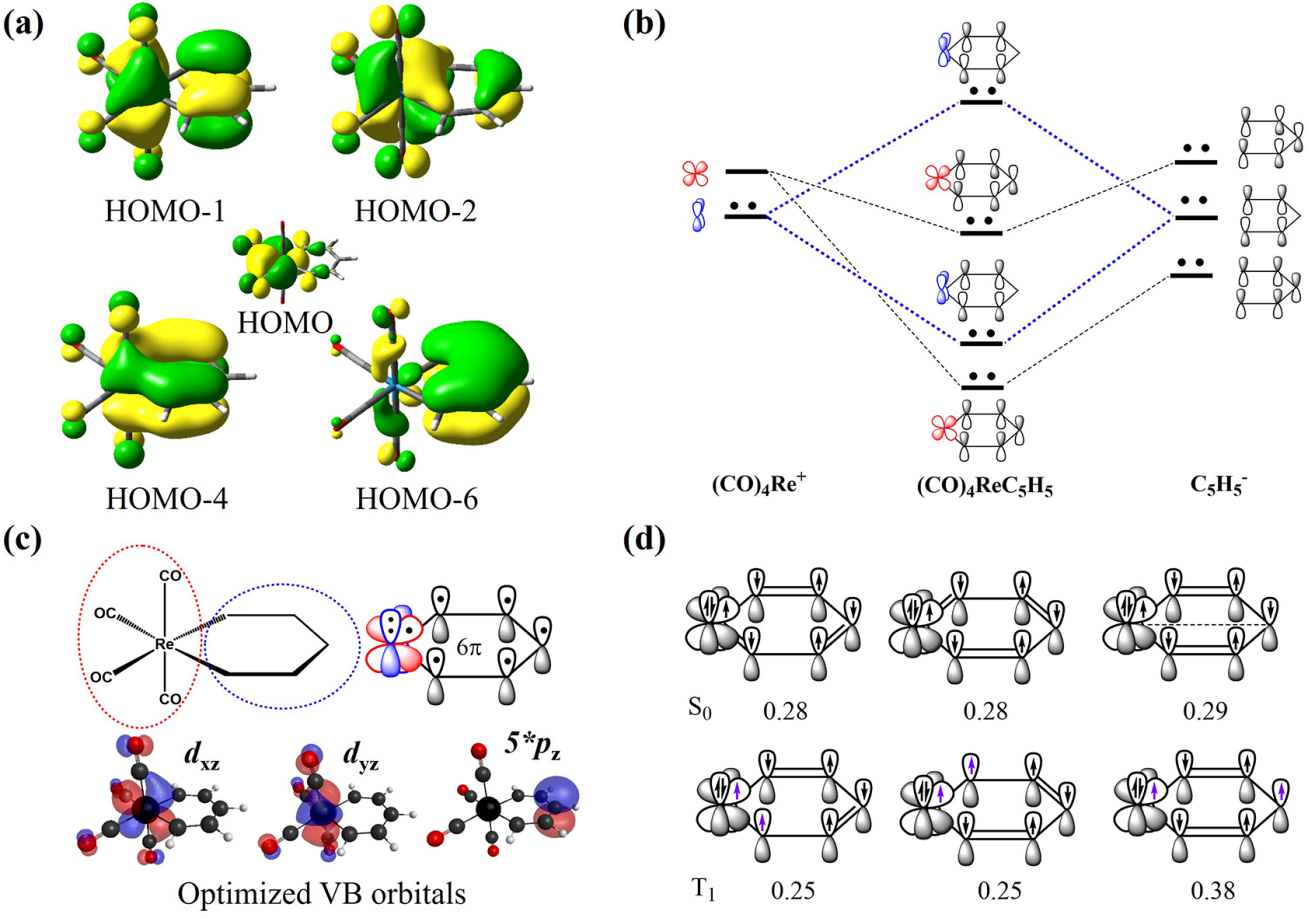
(a) The four occupied π-CMO and HOMO with isovalue = 0.03 for Re­[C_5_H_5_]­(CO)_4_. (b) Schematic representation of a possible π-orbital interaction in rhenabenzene. (c) VBSCF (8e,7o) calculation reveals that the *d*_*yz*_ orbital is doubly occupied, while the *d*_*xz*_ orbital is responsible for the Hückel 6π aromaticity. (d) The three major VB structures for the S_0_ and T_1_ states.

To gain a deeper understanding of the electron delocalization, we conducted VBSCF­(8e,7o) calculations, where the seven active orbitals consist of the *d*_*yz*_ and *d*_*xz*_ orbitals from the (CO)_4_Re fragment and five *p*_*z*_ orbitals strictly localized in each CH group. The remaining orbitals are localized on either (CO)_4_Re or C_5_H_5_, while the two σ orbitals between (CO)_4_Re and C_5_H_5_ are delocalized across the entire system. Due to the delocalization of the *d*_*yz*_ and *d*_*xz*_ orbitals on the (CO)_4_Re fragment, the CO ligands have minor contributions to the optimized VB orbitals, as shown in Figure 5c. Interestingly, the VBSCF­(8e,7o) calculations show that *d*_*yz*_ orbital is doubly occupied; thus, the π system of C_5_H_5_ moiety can only interact with a *d*_*xz*_ orbital. This clearly indicates that rhenabenzene exhibits 6π Hückel aromaticity in the ground state, highlighting again that it is difficult to determine Hückel or Craig aromaticity according to delocalized MOs. Additionally, three major VB structures dominate both the singlet ground (S_0_) state and the triplet excited (T_1_) state, including one Dewar and two Kekulé resonance structures. Since the VB structures in both S_0_ and T_1_ are dominated by the *d*_*xz*_ orbital, they would exhibit Baird antiaromaticity rather than Craig aromaticity in excited states.

To further examine the influence of ligand in metalla-aromaticity, we continue to investigate platinabenzene Pt­[C_5_H_5_]­L with a ligand L = Cp,^117^ which has five occupied π-CMOs according to DFT calculation (see Figure 6a). It is important to note that previous studies have identified the HOMO–9, HOMO–1, and HOMO as the orbitals primarily responsible for aromaticity.^58^ On one hand, platinabenzene exhibits slight nonplanarity, causing HOMO–5 and HOMO–6 to significantly deviate from ideal π-symmetry. On the other hand, the two CMOs indeed represent one doubly occupied *d*_*yz*_ orbital and one doubly occupied π orbital of the Cp ligand, as evidenced by the following VB analysis. A VBSCF­(10e,8o) calculation with ten active electrons and eight active orbitals was conducted. In addition to the two metal *d* orbitals and five carbon *p*_*z*_ orbitals of C_5_H_5_ moiety, one of the π orbitals in L = Cp ligand (as shown in Figure 6c) can also participate in the aromaticity through conjugation with the *d*_*xz*_ orbital, as evidenced by the HOMO and HOMO–5. Similarly, the system is divided into CpPt and C_5_H_5_ fragments. In this regard, the two *d* orbitals and the π orbital of the ligand are localized on the CpPt fragment, while the five *p*_*z*_ orbitals remain localized on each CH group.

**Figure 6:**
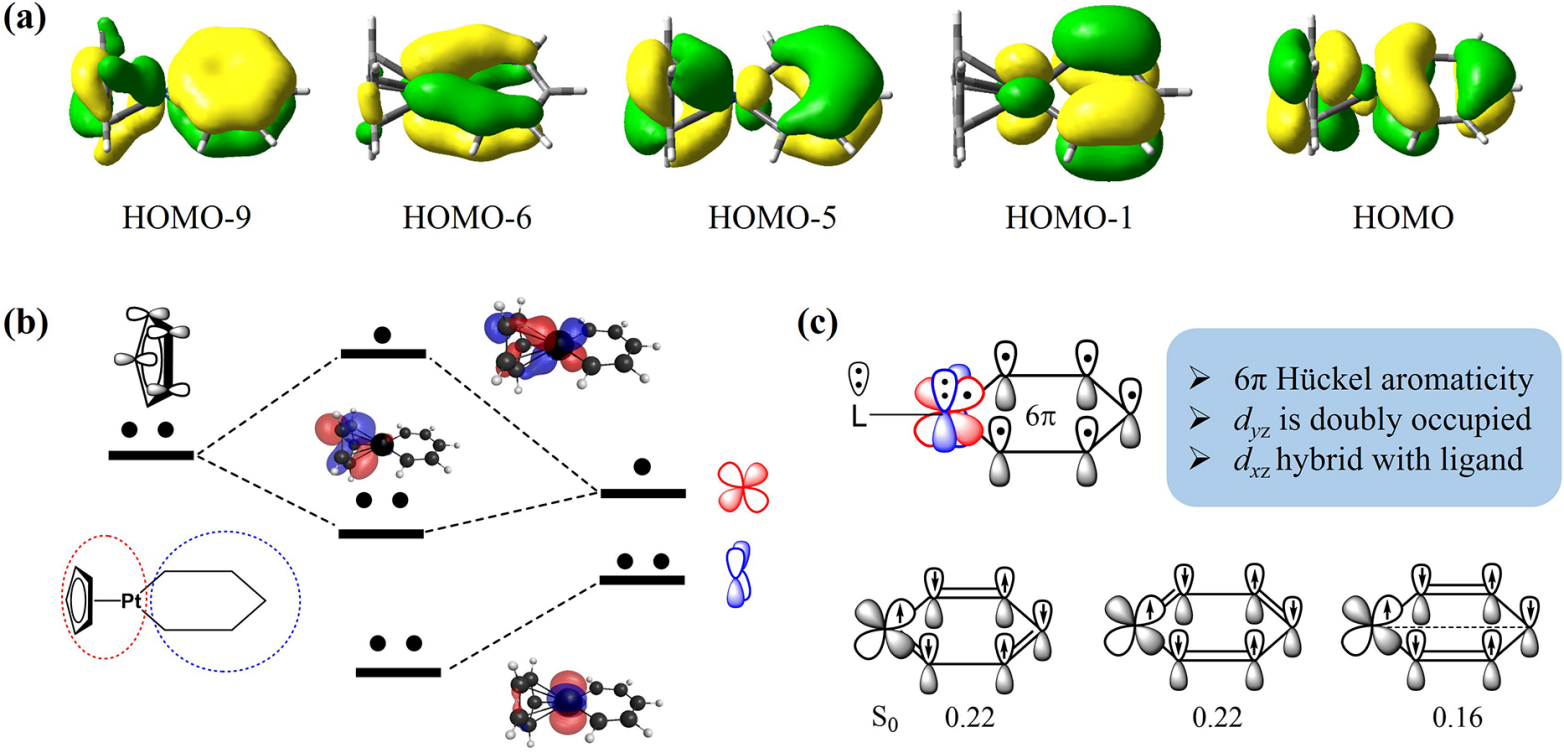
(a) The five occupied π-CMOs with isovalue = 0.03 for Pt­[C_5_H_5_]­Cp. (b) VBSCF (10e,8o) calculation reveals that the *d*_*yz*_ orbital is doubly occupied, while the *d*_*xz*_ orbital or a hybrid orbital between the *d*_*xz*_ and π orbitals of Cp is responsible for the Hückel 6π aromaticity. (c) Three major VB structures for the singlet ground state, where the occupied *d*_*yz*_ and π orbitals of Cp are omitted.

As depicted in Figure 6b, the optimized VB orbitals in the CpPt fragment show that the *d*_*xz*_ orbital can mix with one π orbital of the Cp ligand, resulting in a doubly occupied π orbital primarily localized on the Cp ligand and a singly occupied *d*_*xz*_ orbital. Since the *d*_*yz*_ is doubly occupied, the C_5_H_5_ moiety would interact with *d*_*xz*_ orbital or a hybrid orbital mixed by the *d*_*xz*_ orbital and π orbital of L. As a consequence, the platinabenzene exhibits 6π rather than 10π Hückel aromaticity in the ground state, with an ISE of 24.1 kcal/mol reported in the literature.^60^ Since the *d*_*yz*_ orbital is doubly occupied, platinabenzene is not expected to exhibit Craig excited-state aromaticity.

Given that this work focuses on Craig excited-state aromaticity, it is not necessary to study every reported metallabenzene due to their distinct ligand environments. In other metallabenzenes, such as osmabenzene Os­[C_5_H_5_COS­(PPh_3_)_2_],^118^ iridabenzene Ir­[C_5_H_5_(Me-2,4)­(PEt_3_)_3_],^119^ and Ru­[C_5_H_5_(PPh_3_-2,4)­(PPh_3_)_2_Cl_2_],^120^ the ancillary ligand Cl, PEt_3_, or PPh_3_ may also contribute to the cyclic electron delocalization within the MC_5_H_5_ ring mediated by the metal center. Despite varied ligand complexity, we establish two key principles to understand the aromatic behavior in metalla­benzenes: (1) the *d*_*yz*_ orbital tends to be occupied prior to the *d*_*xz*_ orbital, which is also observed in osmabenzene and related systems;^121^ (2) the C_5_H_5_ moiety would interact with the *d*_*xz*_ orbital or hybrid orbitals involving both the metal center and its ligands. Accordingly, LTM-based metallabenzenes usually exhibit Hückel aromaticity in the ground state and Baird (anti)­aromaticity in the excited states. This is consistent with the findings in the literature that osmabenzene molecules in both ground and excited states exhibit dominant π-Hückel (anti)­aromaticity.^41^

Since *ab initio* VB theory adopts a bottom-up strategy to construct molecular wave functions, it can effectively analyze the bonding nature of a specific system based on its unique characteristics. Therefore, VB methods can be applied to study metallabenzenes of interest, providing insight into the exact number of π-electrons and the corresponding orbitals responsible for aromaticity.

## Conclusions

In this work, we report that ETM-based metallabenzenes can exhibit Craig aromaticity in the lowest singlet and triplet ππ* excited states with 6π electrons, while their LTM counterparts typically display Baird (anti)­aromaticity. Moreover, we found that the excited-state aromaticity in metallacycles exhibits a mixture of Hückel and Craig characteristics, and *ab initio* VB theory provides a straightforward method for determining which of them governs the aromaticity. Specifically, VB methods reveal that the *d*_*yz*_ orbital with a phase change dominates the π_*d‑p*_ electron delocalization in the excited states of ETM-based metallabenzenes, while it is doubly occupied in LTM-based metallabenzenes. Consequently, the LTM-based metallabenzenes usually exhibit Hückel or Baird (anti)­aromaticity through the interaction between the C_5_H_5_ moiety and either the *d*_*xz*_ orbital of the metal center or a hybrid orbital between the *d*_*xz*_ orbital and related orbitals from ligands. In other words, the prerequisite for Craig excited-state aromaticity is the singly occupied or unoccupied *d*_*xz*_ orbital. The discovery of Craig excited-state aromaticity with [4*n*+2] π electrons opens new avenues for the development of a novel class of aromatic metallacycles and significantly enriches the concept of aromaticity.

## Supplementary Material



## Notes

Methodology for VB theory, supplemental figures and tables, and xyz coordinates of optimal geometries for all studied structures (PDF)
