# Supplementary material for: Craig Excited-State Aromaticity in Metallabenzenes: How, When, and Why?
Source: J Am Chem Soc. 2026 Feb 12;148(7):7044–55. doi: 10.1021/jacs.5c18055 (PMC12951443; doi:10.1021/jacs.5c18055)
Supplement: Supplementary file 1 [file ja5c18055_si_001.pdf]

Supporting information for

Craig Excited-State Aromaticity in Metallabenzenes: How, When, and Why?

Xuhui Lin*[1], Mingyang Wei,[1] Yirong Mo*[2]

[1] Hunan Key Laboratory of Super Microstructure and Ultrafast Process, School of Physics, Central South University, Changsha, Hunan 410083, China;

[2] Department of Nanoscience, Joint School of Nanoscience and Nanoengineering, University of North Carolina at Greensboro, Greensboro, NC 27401, United States.

E-mails: xuhui.lin@csu.edu.cn (XL); y_mo3@uncg.edu (YM)

Contents

1. Methodology for valence bond theory....................................,............................S2
2. Table S1 and S2...,... S4
3. Table S3 and S4...,... S5
4. Table S5...,... S6
5. Figure S1 and S2 .... S7
6. Figure S3 ....S8
7. Figure S4 and S5 ....…… S9
8. Figure S6 .... S10
9. xyz coordinates for Optimal Geometries S11

Methodology for valence bond (VB) theory

In VB method, a molecular structure is described with one or a few important resonance structures. Accordingly, the many-electron wave function Ψ is expanded with a set of Heitler-London-Slater-Pauling (HLSP) functions which are used to represent the resonance (Lewis) structures, also called VB structures

$$\Psi = \sum_K C_K \Phi_K \quad (1)$$

where the HSLP Φ_K is defined as

$$\Phi_K = M_K \hat{A}(\varphi_{1,2}\varphi_{3,4}\cdots\varphi_{2n-1,2n}) \quad (2)$$

In the above equation, M_K is the normalization constant, $\hat{A}$ is the antisymmetrizer and $\varphi_{2i-1,2i}$ is a bond function composed of non-orthogonal orbitals ϕ_{2i-1} and ϕ_{2i} (or a lone pair if ϕ_{2i-1}=ϕ_{2i}) as

$$\phi_{2i-1,2i} = \hat{A}\{\varphi_{2i-1}\varphi_{2i}[\alpha(i)\beta(j) - \beta(i)\alpha(j)]\} \quad (3)$$

In VB self-consistent field (VBSCF) procedure, both the VB orbitals and structural coefficients are optimized simultaneously to minimize the total energy. VB structural weights can be evaluated by the following Coulson-Chirgwin formula

$$W_K = C_K^2 + C_K \sum_{L \neq K} M_{KL} C_L \quad (4)$$

Block-localized wavefunction (BLW) method. We note that a HSLP (Eq. 2) can be expanded to a combination of 2^n Slater determinants. To combine the advantages of both MO and VB theories, we proposed the BLW method where a BLW represents a unique electron-localized diabatic state (usually the most stable resonance state). The fundamental assumption is that all electrons and primitive basis functions (χ) can be divided into k subgroups (blocks), and each MO is block-localized and expanded in only one block. Assuming that there are m_i basis and n_i electrons for block i, we can express block-localized MOs for this block as

$$\varphi_j^i = \sum_{\mu=1}^{m_i} c_{j\mu}^i \chi_\mu^i \quad (5)$$

Subsequently, the BLW for a closed-shell is defined using a Slater determinant as

$$\Psi(\text{BLW}) = \left|(\varphi_1^1)^2(\varphi_2^1)^2\cdots(\varphi_{n_1/2}^1)^2\cdots(\varphi_{n_i/2}^i)^2\cdots(\varphi_{n_k/2}^k)^2\right\rangle \qquad = \hat{A}[\Phi_1\cdots\Phi_i\cdots\Phi_k]$$

(6)

Orbitals in the same subspace are subject to the orthogonality constraint as in the MO theory, but orbitals from different subspaces are nonorthogonal as in the VB theory. Since Eq. 6 is self-consistently optimized by minimizing its expectation energy, all local orbitals are thus optimal. The BLW method is available at the DFT level with the geometry optimization and spectra computation capabilities.

Table **S1**. Optimal bond distances (in Å) for T_1 and S_1 states of with TDDFT and CASSCF methods.

Mol	TDDFT (T1)			CASSCF(T1)			CASSCF(S1)		
	M–C_1	C_1–C_2	C_3–C_4	M–C_1	C_1–C_2	C_3–C_4	M–C_1	C_1–C_2	C_3–C_4
$TiHC_5H_5$	2.053	1.380	1.410	2.066	1.376	1.423	2.094	1.383	1.425
ScC_5H_5	2.097	1.389	1.413	2.240	1.381	1.429	2.234	1.387	1.429
YC_5H_5	2.228	1.392	1.412	2.351	1.382	1.426	2.339	1.390	1.427
LaC_5H_5	2.327	1.390	1.411	2.506	1.379	1.424	2.494	1.385	1.425
AcC_5H_5	2.410	1.393	1.413	2.547	1.363	1.428	2.562	1.359	1.435

Table **S2**. Adiabatic excitation energies (in kcal/mol) for S_1 (ΔE_{SS*}) and T_1 (ΔE_{ST*}) states at the DFT, CASSCF and VBSCF levels.

Mol	TDDFT		CASSCF (6e, 7o)		VBSCF (6e, 7o)	
	ΔE_{SS*}	ΔE_{ST*}	ΔE_{SS*}	ΔE_{ST*}	ΔE_{SS*}	ΔE_{ST*}
$TiHC_5H_5$	21.45	-1.21 (6.73)	1.86	3.19	3.84	10.65
ScC_5H_5	32.71	21.97 (9.30)	8.76	10.06	9.99	11.15
YC_5H_5	37.36	27.60 (23.35)	13.52	14.70	12.94	16.02
LaC_5H_5	37.82	28.75 (24.14)	10.90	11.76	13.72	14.83
AcC_5H_5	38.95	30.56 (25.12)	29.17	26.69	27.32	26.36

The data in the bracket are obtained by UDFT for T_1 states

Table **S3**. Optimal bond distances (in Å), NICS(1)$_{zz}$ (in ppm), induced ring current density (J^{ind}, in nA/T) for the lowest $\pi\pi$* triplet state at the UPBE0 level.

Mol	M−$C_{1/5}$	C_1−C_2	C_3−C_4	NICS(1)$_{zz}$	J^{ind}
$ClTiC_5H_5$	2.046	1.372	1.414	-20.8	8.1
$CH_3TiC_5H_5$	2.056	1.374	1.417	-22.2	8.1
$ZrHC_5H_5$	2.161	1.385	1.418	-25.2	10.1
$HfHC_5H_5$	2.150	1.389	1.418	-22.6	10.1
$ThHC_5H_5$	2.309	1.402	1.424	-20.5	10.0

Table **S4**. The weights for the three major VB structures from VBSCF(6e, 7o) calculations with full VB structure, all covalent VB structures and only three major VB structures.

Mol		S_0			S_1			T_1		
		D	K	K	D	K	K	D	K	K
$TiHC_5H_5$	Full	0.144	0.121	0.121	0.165	0.130	0.130	0.163	0.100	0.100
	Cov	0.331	0.244	0.244	0.343	0.257	0.257	0.350	0.196	0.196
ScC_5H_5	Full	0.143	0.122	0.122	0.159	0.133	0.133	0.166	0.102	0.102
	Cov	0.351	0.244	0.244	0.327	0.267	0.267	0.369	0.202	0.202
YC_5H_5	Full	0.080	0.107	0.107	0.132	0.131	0.131	0.150	0.091	0.091
	Cov	0.267	0.258	0.258	0.273	0.262	0.2262	0.356	0.190	0.190
LaC_5H_5	Full	0.087	0.108	0.108	0.138	0.129	0.129	0.153	0.095	0.095
	Cov	0.291	0.256	0.256	0.286	0.262	0.262	0.387	0.242	0.242
AcC_5H_5	Full	0.071	0.098	0.098	0.143	0.131	0.131	0.156	0.096	0.096
	Cov	0.311	0.266	0.266	0.322	0.272	0.272	0.355	0.202	0.202

Table **S5**. NICS(1)$_{zz}$ values for MHC_5H_5 and MC_5H_5, their saturated analogues $H_2MC_5H_{10}$ and HMC5H_{10} as well as the corresponding deviations (D = NICS (MHC_5H_5)-NICS($H_2MC_5H_{10}$)).

Mol	NICS(1)$_{zz}$	D
$TiHC_5H_5$	-26.2	-21.2
$TiH_2C_5H_{10}$	-5.0	
$ZrHC_5H_5$	-25.2	-21.7
$ZrH_2C_5H_{10}$	-3.5	
$HfHC_5H_5$	-22.6	-21.6
$HfH_2C_5H_5$	-1.0	
$ThHC_5H_5$	-20.5	-17.9
$ThH_2C_5H_5$	-2.6	
ScC_5H_5	-33.3	-34.3
$ScHC_5H_{10}$	1.0	
YC_5H_5	-28.3	-30.0
YHC_5H_{10}	1.7	
LaC_5H_5	-22.3	-24.4
$LaHC_5H_{10}$	2.1	
AcC_5H_5	-24.2	-27.8
$AcHC_5H_{10}$	3.6	

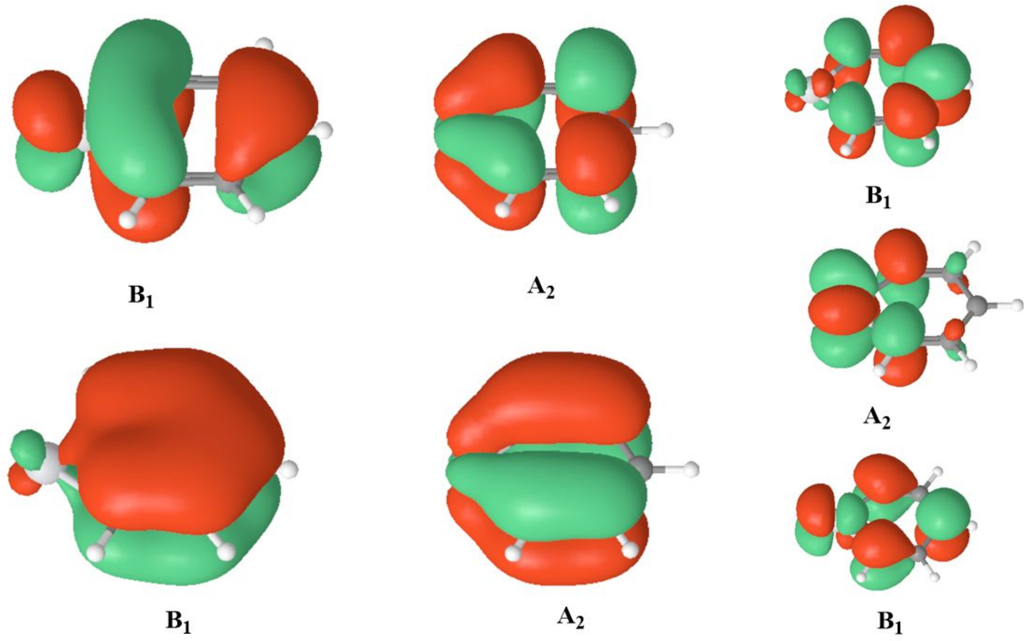

Figure **S1**. The seven active molecular orbitals used in the CASSCF(6,7) calculations.

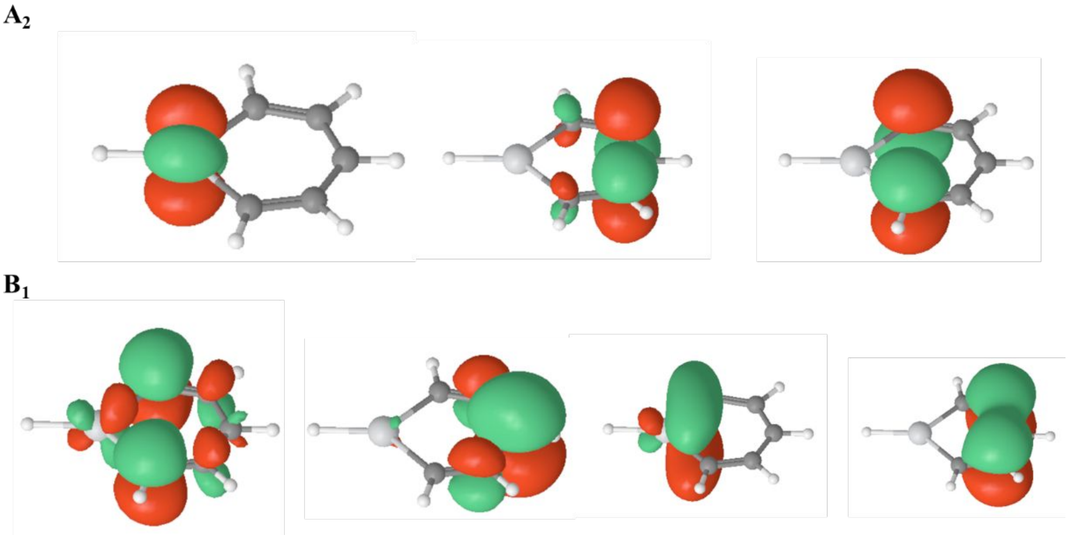

Figure **S2**. The localized active molecular orbitals with CASSCF(6,7) by using Pipek–Mezey localization.

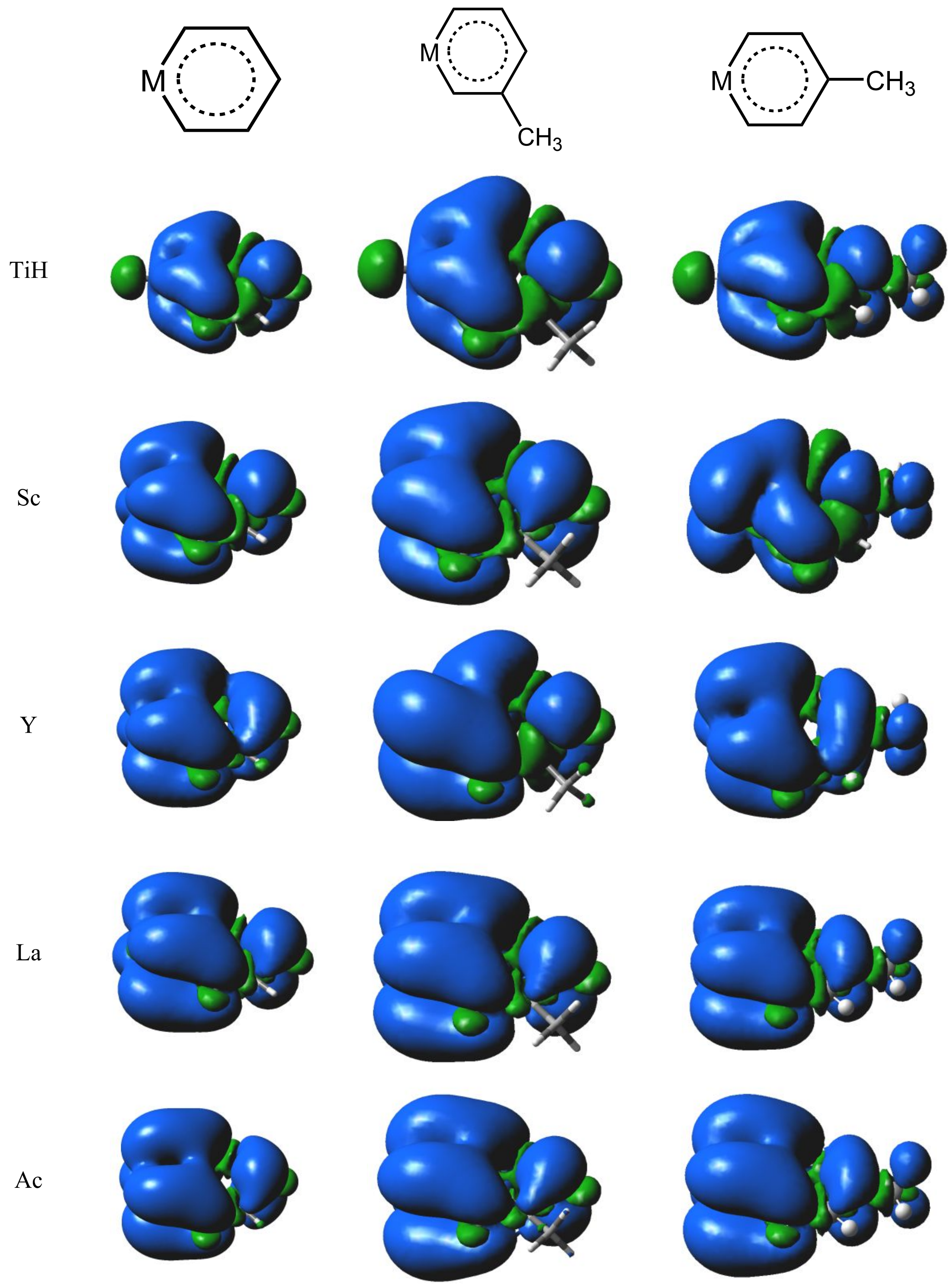

Figure **S3**. The spin density (isovalue = 0.0004 a.u.) for ETM-based metallabenzenes and their corresponding delocalized isomer II used to evaluate ISE.

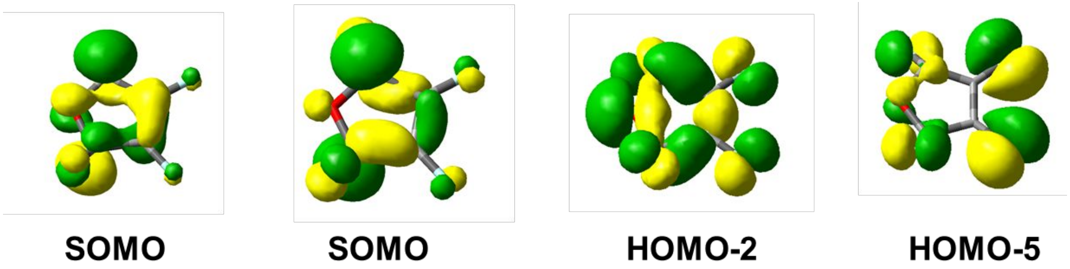

Figure **S4**. The CMOs for the T_1 state of twisted C_4OF_4 with Heilbronner-Möbius aromaticity.

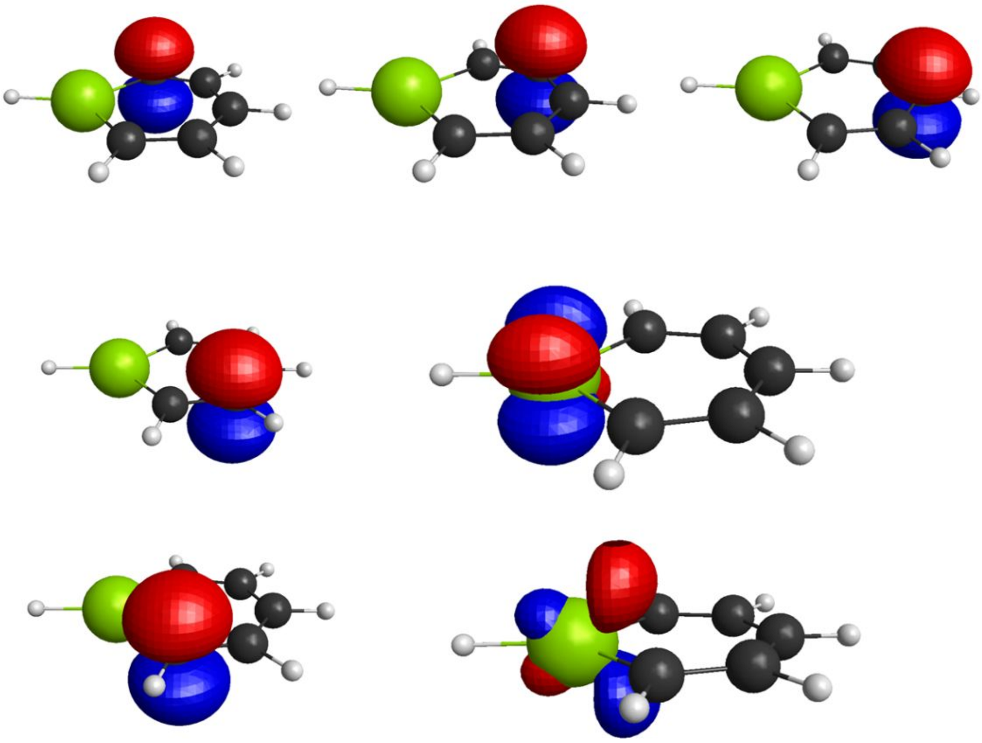

Figure **S5**. The seven active orbitals used in the VBSCF(6,7) calculations.

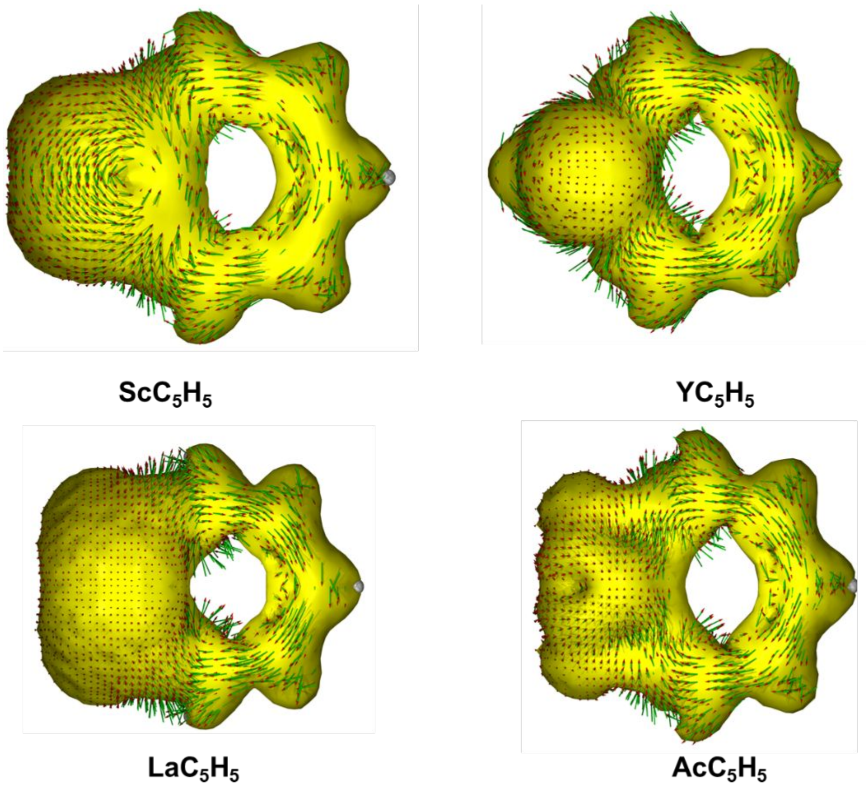

Figure **S6**. The ACID plots for the T_1 states of MC_5H_5 (M = Sc, Y, La and Ac).

xyz coordinates for Optimal Geometries

T_1-$TiHC_5H_5$-CASPT2

C	0.00000000	1.51738189	0.03176599
C	0.00000000	-1.51738189	0.03176599
C	0.00000000	1.27582500	-1.32720790
C	0.00000000	-1.27582500	-1.32720790
C	0.00000000	0.00000000	-1.94861260
H	0.00000000	2.57705045	0.30306308
H	0.00000000	-2.57705045	0.30306308
H	0.00000000	2.11659212	-2.02003632
H	0.00000000	-2.11659212	-2.02003632
H	0.00000000	0.00000000	-3.03409340
Ti	0.00000000	0.00000000	1.39119776
H	0.00000000	0.00000000	3.16846515

S_1-$TiHC_5H_5$-CASPT2

C	0.00000000	1.53387166	0.04282618
C	0.00000000	-1.53387166	0.04282618
C	0.00000000	1.27943686	-1.32022482
C	0.00000000	-1.27943686	-1.32022482
C	0.00000000	0.00000000	-1.94103908
H	0.00000000	2.59311528	0.31074981
H	0.00000000	-2.59311528	0.31074981
H	0.00000000	2.11692309	-2.01672531
H	0.00000000	-2.11692309	-2.01672531
H	0.00000000	0.00000000	-3.02609626
Ti	0.00000000	0.00000000	1.37194604

H	0.00000000	0.00000000	3.14310519

S_0-TiHC_5H_5-PBE0

C	-1.45205400	-0.24554600	0.75279200
C	-1.27161600	0.95021800	0.08604100
C	-0.00001300	1.37981400	-0.41474200
C	1.27159800	0.95023900	0.08604100
C	1.45205400	-0.24551800	0.75279900
H	-2.41649900	-0.51779900	1.16725200
H	-2.12110400	1.57524900	-0.18550700
H	-0.00002000	2.18656900	-1.13604200
H	2.12107600	1.57528300	-0.18550700
H	2.41650400	-0.51775700	1.16725500
Ti	0.00000800	-0.84831100	-0.32789200
H	0.00005200	-2.37394600	-1.19141400

T_1-TiHC_5H_5-PBE0 (UDFT)

C	0.00000000	1.53671614	0.02805127
C	0.00000000	1.27594114	-1.31995140
C	0.00000000	0.00000000	-1.93242898
C	0.00000000	-1.27594114	-1.31995140
C	-0.00000000	-1.53671614	0.02805127
H	0.00000000	2.59946086	0.28790172
H	0.00000000	2.11063111	-2.02196010
H	0.00000000	0.00000000	-3.01896488
H	0.00000000	-2.11063111	-2.02196010
H	-0.00000000	-2.59946086	0.28790172
Ti	-0.00000000	-0.00000000	1.38297919
H	-0.00000000	-0.00000000	3.15891493

S_1-TiHC_5H_5-PBE0 (TDDFT)

C	0.00000000	1.52952900	0.06950900
C	0.00000000	1.27117600	-1.29054200
C	0.00000000	0.00000000	-1.90137400
C	0.00000000	-1.27117600	-1.29054200
C	0.00000000	-1.52952900	0.06950900
H	0.00000000	2.59192700	0.32981200
H	0.00000000	2.10833200	-1.98883600
H	0.00000000	0.00000000	-2.98770000
H	0.00000000	-2.10833200	-1.98883600
H	0.00000000	-2.59192700	0.32981200
Ti	0.00000000	0.00000000	1.38169500
H	0.00000000	0.00000000	3.15126600

T_1-ClTiC_5H_5-PBE0

C	0.00000000	1.51553959	-0.77525090
C	0.00000000	1.27119594	-2.12518343
C	0.00000000	-0.00000000	-2.74378117
C	0.00000000	-1.27119594	-2.12518343
C	-0.00000000	-1.51553959	-0.77525090
H	0.00000000	2.57406874	-0.50069848
H	0.00000000	2.11325167	-2.81770102
H	0.00000000	-0.00000000	-3.83017495
H	0.00000000	-2.11325167	-2.81770102
H	-0.00000000	-2.57406874	-0.50069848
Ti	-0.00000000	0.00000000	0.59955587
Cl	-0.00000000	0.00000000	2.85556729

S_1-ClTiC_5H_5-PBE0

C	0.00000000	1.50347700	-0.76581400
C	0.00000000	1.26631600	-2.12723500
C	0.00000000	0.00000000	-2.74483200
C	0.00000000	-1.26631600	-2.12723500
C	0.00000000	-1.50347700	-0.76581400
H	0.00000000	2.56126600	-0.48836300
H	0.00000000	2.11153500	-2.81508600
H	0.00000000	0.00000000	-3.83111100
H	0.00000000	-2.11153500	-2.81508600
H	0.00000000	-2.56126600	-0.48836300
Ti	0.00000000	0.00000000	0.57960500
Cl	0.00000000	0.00000000	2.83283100

T_1-CH3TiC_5H_5-PBE0

C	-0.02672626	-0.40293634	1.55832993
C	-0.02672626	-1.74845693	1.27996569
C	-0.03912265	-2.35520514	0.00000000
C	-0.02672626	-1.74845693	-1.27996569
C	-0.02672626	-0.40293634	-1.55832993
H	0.01709968	-0.16060525	2.62343761
H	-0.00250355	-2.45933205	2.10738845
H	-0.03809694	-3.44186020	0.00000000
H	-0.00250355	-2.45933205	-2.10738845
H	0.01709968	-0.16060525	-2.62343761
Ti	-0.11495464	0.93534192	-0.00000000
C	0.33911810	2.98800587	-0.00000000
H	-0.02785399	3.51917717	0.88747493
H	1.43507217	3.08577301	-0.00000000
H	-0.02785399	3.51917717	-0.88747493

S_1-CH3TiC_5H_5-PBE0

C	-0.00506800	-0.37630600	1.55669000
C	-0.00506800	-1.73360800	1.27566000
C	-0.01500300	-2.33800600	0.00000000
C	-0.00506800	-1.73360800	-1.27566000
C	-0.00506800	-0.37630600	-1.55669000
H	0.04576100	-0.13691800	2.62159600
H	0.01484000	-2.44216100	2.10457500
H	-0.01157600	-3.42434100	0.00000000
H	0.01484000	-2.44216100	-2.10457500
H	0.04576100	-0.13691800	-2.62159600
Ti	-0.09225600	0.90675100	0.00000000
C	0.20707100	2.97819500	0.00000000
H	-0.20157000	3.47632600	0.88838700
H	1.29237300	3.15915100	0.00000000
H	-0.20157000	3.47632600	-0.88838700
Bq	-0.02125517	-0.94184717	0.00000000

S_0-ScC_5H_5-PBE0

C	0.00000000	1.48378172	0.10942908
C	0.00000000	1.23532442	-1.26044846
C	0.00000000	-0.00000000	-1.90975544
C	0.00000000	-1.23532442	-1.26044846
C	-0.00000000	-1.48378172	0.10942908
H	0.00000000	2.54742006	0.37959934
H	0.00000000	2.10284577	-1.92541162
H	0.00000000	-0.00000000	-2.99410162
H	0.00000000	-2.10284577	-1.92541162

H	-0.00000000	-2.54742006	0.37959934
Sc	-0.00000000	0.00000000	1.49316626

T_1-ScC_5H_5-PBE0

C	0.00000000	1.57909439	0.07775708
C	0.00000000	1.28394514	-1.26872445
C	0.00000000	-0.00000000	-1.86683293
C	0.00000000	-1.28394514	-1.26872445
C	-0.00000000	-1.57909439	0.07775708
H	0.00000000	2.65420965	0.28838392
H	0.00000000	2.10438330	-1.99012026
H	0.00000000	-0.00000000	-2.95445087
H	0.00000000	-2.10438330	-1.99012026
H	-0.00000000	-2.65420965	0.28838392
Sc	-0.00000000	0.00000000	1.51669188

S_1-ScC_5H_5-PBE0

C	0.00000000	1.57050300	0.09176100
C	0.00000000	1.27743000	-1.26122900
C	0.00000000	0.00000000	-1.86481600
C	0.00000000	-1.27743000	-1.26122900
C	0.00000000	-1.57050300	0.09176100
H	0.00000000	2.64282400	0.31209700
H	0.00000000	2.10358400	-1.97579200
H	0.00000000	0.00000000	-2.95158600
H	0.00000000	-2.10358400	-1.97579200
H	0.00000000	-2.64282400	0.31209700
Sc	0.00000000	0.00000000	1.50007000

S_0-YC_5H_5-PBE0

C	0.00000000	1.51774262	-0.34668896
C	0.00000000	1.24182522	-1.71290257
C	0.00000000	0.00000000	-2.35203116
C	0.00000000	-1.24182522	-1.71290257
C	-0.00000000	-1.51774262	-0.34668896
H	0.00000000	2.59191948	-0.11430422
H	0.00000000	2.09822557	-2.39315533
H	0.00000000	0.00000000	-3.43681783
H	0.00000000	-2.09822557	-2.39315533
H	-0.00000000	-2.59191948	-0.11430422
Y	-0.00000000	-0.00000000	1.21228262

T_1-YC_5H_5-PBE0

C	0.00000000	1.57934975	-0.38042725
C	0.00000000	1.28400579	-1.73265870
C	0.00000000	-0.00000000	-2.32722970
C	0.00000000	-1.28400579	-1.73265870
C	-0.00000000	-1.57934975	-0.38042725
H	0.00000000	2.65671615	-0.17192691
H	0.00000000	2.10500780	-2.45258580
H	0.00000000	-0.00000000	-3.41523592
H	0.00000000	-2.10500780	-2.45258580
H	-0.00000000	-2.65671615	-0.17192691
Y	-0.00000000	0.00000000	1.23037617

S_1-YC_5H_5-PBE0

C	0.00000000	1.58129200	-0.37744100
C	0.00000000	1.27974500	-1.72932800
C	0.00000000	0.00000000	-2.32939200
C	0.00000000	-1.27974500	-1.72932800
C	0.00000000	-1.58129200	-0.37744100
H	0.00000000	2.65803900	-0.17036000
H	0.00000000	2.10257500	-2.44792000
H	0.00000000	0.00000000	-3.41634800
H	0.00000000	-2.10257500	-2.44792000
H	0.00000000	-2.65803900	-0.17036000
Y	0.00000000	0.00000000	1.22847400

Planar [LaC_5H_5]

C	0.00000000	1.44382267	-0.68813007
C	0.00000000	1.23394846	-2.06223698
C	0.00000000	0.00000000	-2.71694855
C	-0.00000000	-1.23394846	-2.06223698
C	-0.00000000	-1.44382267	-0.68813007
H	-0.00000000	2.51001499	-0.39359207
H	0.00000000	2.10654931	-2.72117795
H	0.00000000	0.00000000	-3.80164863
H	-0.00000000	-2.10654931	-2.72117795
H	-0.00000000	-2.51001499	-0.39359207
La	0.00000000	-0.00000000	1.04100499

S_0-LaC_5H_5-PBE0

C	-0.00000000	1.54371567	-0.73571150
C	-0.00000000	1.27849440	-2.08981108
C	0.00000000	-0.00000000	-2.69438386
C	-0.00000000	-1.27849440	-2.08981108

C	-0.00000000	-1.54371567	-0.73571150
H	-0.00000000	2.62010512	-0.50227595
H	-0.00000000	2.10745209	-2.80109290
H	0.00000000	-0.00000000	-3.78236310
H	-0.00000000	-2.10745209	-2.80109290
H	-0.00000000	-2.62010512	-0.50227595
La	-0.00000000	0.00000000	1.06073114

S_1-LaC_5H_5-PBE0

C	0.00000000	1.53559100	-0.73559200
C	0.00000000	1.27261400	-2.09120400
C	0.00000000	0.00000000	-2.70323800
C	0.00000000	-1.27261400	-2.09120400
C	0.00000000	-1.53559100	-0.73559200
H	0.00000000	2.61136800	-0.50031300
H	0.00000000	2.10573400	-2.79831300
H	0.00000000	0.00000000	-3.79029100
H	0.00000000	-2.10573400	-2.79831300
H	0.00000000	-2.61136800	-0.50031300
La	0.00000000	0.00000000	1.06190400

S_0-AcC_5H_5-PBE0

C	0.00000000	1.54493188	-0.99293846
C	-0.00000000	1.24857607	-2.35374031
C	-0.00000000	0.00000000	-2.98369807
C	-0.00000000	-1.24857607	-2.35374031
C	-0.00000000	-1.54493188	-0.99293846
H	0.00000000	2.62473800	-0.77937239
H	0.00000000	2.09412571	-3.04843661

H	-0.00000000	0.00000000	-4.06876776
H	-0.00000000	-2.09412571	-3.04843661
H	-0.00000000	-2.62473800	-0.77937239
Ac	0.00000000	-0.00000000	0.78412044

T_1-AcC_5H_5-PBE0

C	0.00000000	1.60163753	-1.04907262
C	0.00000000	1.28743417	-2.39470003
C	0.00000000	0.00000000	-2.98293901
C	-0.00000000	-1.28743417	-2.39470003
C	-0.00000000	-1.60163753	-1.04907262
H	0.00000000	2.68356334	-0.85802239
H	0.00000000	2.09909457	-3.12618140
H	0.00000000	0.00000000	-4.07092474
H	-0.00000000	-2.09909457	-3.12618140
H	-0.00000000	-2.68356334	-0.85802239
Ac	-0.00000000	-0.00000000	0.80069931

S_1-AcC_5H_5-PBE0

C	0.00000000	1.59991100	-1.04830800
C	0.00000000	1.28281200	-2.39345200
C	0.00000000	0.00000000	-2.98792500
C	0.00000000	-1.28281200	-2.39345200
C	0.00000000	-1.59991100	-1.04830800
H	0.00000000	2.68173400	-0.85962700
H	0.00000000	2.09712300	-3.12265800
H	0.00000000	0.00000000	-4.07496000
H	0.00000000	-2.09712300	-3.12265800
H	0.00000000	-2.68173400	-0.85962700
Ac	0.00000000	0.00000000	0.80076600

S_0-Re[C_5H_5]$(CO)_4$-PBE0

O	0.00000000	2.36770974	2.28307721
C	-0.00000000	1.24930109	-2.69872037
C	-0.00000000	-0.00000000	-3.32221349
C	-0.00000000	1.42835464	-1.33521388
C	-0.00000000	-1.24930109	-2.69872037
C	-0.00000000	-1.42835464	-1.33521388
C	0.00000000	1.48336170	1.56011712
H	-0.00000000	0.00000000	-4.40978672
H	-0.00000000	2.48912344	-1.07167142
H	-0.00000000	-2.11569825	-3.35540660
H	-0.00000000	-2.48912344	-1.07167142
H	0.00000000	2.11569825	-3.35540660
C	2.00213110	-0.00000000	0.27861395
C	-2.00213110	0.00000000	0.27861395
C	-0.00000000	-1.48336170	1.56011712
O	3.13776487	-0.00000000	0.30870257
O	-3.13776487	0.00000000	0.30870257
O	-0.00000000	-2.36770974	2.28307721
Re	0.00000000	0.00000000	0.24094914

T_1-Re[C_5H_5]$(CO)_4$-PBE0

O	0.00000000	2.28756012	2.49137531
C	0.00000000	1.25984082	-2.58783475
C	-0.00000000	-0.00000000	-3.21901146
C	-0.00000000	1.49654352	-1.24053519
C	-0.00000000	-1.25984082	-2.58783475
C	-0.00000000	-1.49654352	-1.24053519
C	0.00000000	1.46837318	1.70590031
H	-0.00000000	-0.00000000	-4.30381389
H	0.00000000	2.54889968	-0.95833874
H	0.00000000	-2.12098395	-3.25321297
H	-0.00000000	-2.54889968	-0.95833874
H	0.00000000	2.12098395	-3.25321297
C	1.98780906	-0.00000000	0.04037631
C	-1.98780906	0.00000000	0.04037631
C	-0.00000000	-1.46837318	1.70590031
O	3.10179899	-0.00000000	-0.16868096
O	-3.10179899	0.00000000	-0.16868096
O	-0.00000000	-2.28756012	2.49137531
Re	0.00000000	0.00000000	0.26483995

S_0- Pt[C_5H_5]Cp -PBE0

C	-2.06173223	-0.37250449	1.14747656
C	-2.06173223	0.98438686	0.70675872
C	-2.06173223	0.98438686	-0.70675872
C	-2.06173223	-0.37250449	-1.14747656
C	-2.06957357	-1.20592107	0.00000000
H	-2.06011588	-0.70348874	2.17361264
H	-2.05932824	1.85412362	1.34421611
H	-2.05932824	1.85412362	-1.34421611
H	-2.06011588	-0.70348874	-2.17361264
H	-2.06809179	-2.28437954	0.00000000
C	1.28717596	-1.34137565	0.00000000
C	1.28401032	1.33924409	0.00000000
H	0.91614431	-2.36812997	0.00000000
H	0.90799201	2.36434325	0.00000000
C	2.65691395	-1.21996480	0.00000000
C	2.65430857	1.22244058	0.00000000
H	3.25777356	-2.12500184	0.00000000
H	3.25252614	2.12920092	0.00000000
C	3.31967860	0.00212651	0.00000000
Pt	-0.09930846	-0.00182676	0.00000000
H	4.40509452	0.00329844	0.00000000
